# Nutraceuticals and Diet Supplements in Crohn’s Disease: A General Overview of the Most Promising Approaches in the Clinic

**DOI:** 10.3390/foods11071044

**Published:** 2022-04-04

**Authors:** Barbara De Conno, Marcella Pesce, Martina Chiurazzi, Marta Andreozzi, Sara Rurgo, Chiara Corpetti, Luisa Seguella, Alessandro Del Re, Irene Palenca, Giuseppe Esposito, Giovanni Sarnelli

**Affiliations:** 1 Department of Clinical Medicine and Surgery, University of Naples “Federico II”, 80131 Naples, Italy; barbara.deconno@gmail.com (B.D.C.); marcella.pesce@unina.it (M.P.); martina.chiurazzi88@gmail.com (M.C.); marta.snpp@gmail.com (M.A.); sararurgo91@gmail.com (S.R.); 2 Department of Pharmacy, University of Naples “Federico II”, 80131 Naples, Italy; 3 Department of Physiology and Pharmacology “V. Erspamer”, “Sapienza” University of Rome, Piazzale Aldo Moro 5, 00185 Rome, Italy; chiara.corpetti@uniroma1.it (C.C.); luisa.seguella@uniroma1.it (L.S.); alessandro.delre@uniroma1.it (A.D.R.); irene.palenca@uniroma1.it (I.P.); giuseppe.esposito@uniroma1.it (G.E.)

**Keywords:** inflammatory bowel diseases, Crohn’s disease, nutraceutical compounds, phytotherapics, palmytoilethanolamide (PEA), lactoferrin, probiotics

## Abstract

Crohn’s disease (CD) is a chronic inflammatory gastrointestinal disorder requiring lifelong medications. The currently approved drugs for CD are associated with relevant side effects and several studies suggest an increased use of nutraceuticals among CD patients, seeking for what is perceived as a more “natural” approach in controlling this highly morbid condition. Nutraceuticals are foods or foods’ components with beneficial health properties that could aid in CD treatment for their anti-inflammatory, analgesic and immunoregulatory activities that come along with safety, high tolerability, easy availability and affordability. Depending on their biological effect, nutraceuticals’ support could be employed in different subsets of CD patients, both those with active disease, as adjunctive immunomodulatory therapies, and/or in quiescent disease to provide symptomatic relief in patients with residual functional symptoms. Despite the increasing interest of the general public, both limited research and lack of education from healthcare professionals regarding their real clinical effectiveness account for the increasing number of patients turning to unconventional sources. Professionals should recognize their widespread use and the evidence base for or against their efficacy to properly counsel IBD patients. Overall, nutraceuticals appear to be safe complements to conventional therapies; nonetheless, little quality evidence supports a positive impact on underlying inflammatory activity.

## 1. Introduction

Inflammatory bowel diseases (IBD), including Crohn’s Disease (CD) and Ulcerative Colitis (UC), are chronic inflammatory gastrointestinal disorders with unknown etiology and a continuously rising incidence, probably resulting from the interaction between genetic and environmental factors [1,2].

The goal of IBD therapy is primarily to induce and maintain disease remission, which means achieving mucosal healing as well as transmural healing, if considering CD along with symptoms’ relief. Unfortunately, in a significant proportion of patients, this result is still difficult to obtain and often requires lifelong treatment [3]. The currently approved drugs, including corticosteroids, immunosuppressants and novel biologic agents, are associated with relevant side effects, prompting patients to often seek alternative or complementary medicine approaches in order to limit over-medicalization. 

Moreover, even when remission is achieved, IBD patients often suffer from overlapping functional irritable bowel syndrome-like (IBS-like) symptoms, such as bloating, abdominal pain and altered bowel frequency (diarrhea and/or constipation), which importantly affect their quality of life. It is estimated that approximately 40% of IBD patients may suffer from chronic IBS-like symptoms and their prevalence in patients with CD is higher than in those with UC [4].

The role of diet and dietary manipulations has gained momentum in the scientific community as a helpful and underappreciated tool to modify the course of underlying chronic inflammation in CD [5]. The most convincing evidence supporting the role of food(s) and/or their components in modifying disease course derives from epidemiological cohort studies linking specific nutrient deficiencies with the increased risk of IBD and the pivotal role of exclusive enteral nutrition as the main induction treatment in active pediatric CD [6].

Not surprisingly, dietary manipulations and supplements, including nutraceuticals, are increasingly gaining attention by the general public as alternative or adjuvant therapies in CD, as they are seen by patients as a more natural approach in long-term IBD care. It has been estimated that IBD patients are more likely to seek out complementary and alternative treatments, with a nearly doubled prevalence as compared to other gastrointestinal disorders (15% vs. 8%, respectively) [7]. This tendency is far greater in CD than UC patients (38% vs. 27%, respectively; *p* = 0.01), according to a Norwegian population-based survey [8]. This interesting aspect might be explained by several factors that differentiate UC and CD patients, with higher risks of surgical complications and disease flares requiring steroids and/or immunomodulators to control intestinal inflammation and a poorer quality of life (QoL) as compared to UC patients. Interestingly, in a recent paper, nutritional status resulted to be one of the main predictors of health-related QoL in a cross-sectional study involving consecutive CD patients, stressing the importance of preventing malnutrition through dietary interventions to improve disease burden in this population of patients [9]. 

The word “nutraceutical” derives from the fusion of the terms “nutrition” and “pharmaceutical”. They are food or foods’ components with beneficial health properties and a lack of significant side effects, which may play a role in the prevention and treatment of different diseases [10]. Depending on the source, the definition of nutraceuticals can be narrowed down to isolated and/or purified plant-derived components or metabolites (e.g., polyphenols) or encompass the broader umbrella definition of any food-derived compound with protective health properties, thus including herbal extracts (phytochemicals), micronutrients (vitamins, peptides and fatty acids) and dietary supplements (probiotics and synbiotics) [11,12]. 

Different mechanisms are thought to be involved in CD’s pathophysiology, including immune dysregulation, oxidative stress, impaired intestinal microbiome, damage and increased permeability of the gastrointestinal mucosal barrier [13,14]. Depending on their biological targets and mechanisms of action, nutraceuticals’ use may be considered in different subsets of CD patients, both in active inflammatory disease and in phases of remission with persisting IBS-like symptoms.

Patients with CD often empirically use herbs or dietary supplements in the attempt of coping better with their chronic condition. Being perceived as harmless, nutraceuticals are often suggested by patients through word of mouth or the Internet based solely on anecdotal evidence. Regretfully, the information found on the Internet about nutraceuticals does not always derive from reliable sources, given the vast financial interests. 

Clinicians often have a dismissive approach on the topic, tending to encourage patients to look for alternative unverified sources, with detrimental consequences, at times. Indeed, herbal dietary supplements have become a relevant concern for regulatory authorities given the increasing number of case reports of serious adverse events, particularly of drug-induced liver injury [15]. 

Furthermore, health safety issues may not only depend on the intrinsic toxicological profile of dietary supplements, but also by inadequate quality control resulting in the contamination and/or adulteration of nutraceuticals, due to the less rigorous regulation compared to the pharmaceutical sector [16].

The aim of this review is to analyze the nutraceuticals currently used in the treatment of CD and to collect findings from both clinical and preclinical trials exploring promising nutraceuticals and their possible applications in active and/or quiescent CD patients to aid clinicians in adequately counselling their patients.

## 2. Phytotherapics

Phytochemicals are herbal chemical compounds, produced by plants primarily to protect themselves from bacterial and fungal infections. Medical plants have been empirically used in the past as traditional remedies, but some phytochemicals are currently under research for their therapeutic role in different pathological conditions, including functional and organic bowel disorders. Among phytochemicals, the family of polyphenols, which are contained in several plants, such as green tea leaves and *Curcuma longa* (curcumin), are of particular interest in the field of IBD for their anti-inflammatory properties that have also been demonstrated in human randomized controlled trials (RCTs) (Table 1).

Green tea polyphenols (GrTP) seem to have anti-inflammatory effects in different chronic inflammatory diseases, including gastrointestinal malignancies, IBD and hepatic and neurodegenerative disorders. GrTP showed a similar efficacy to sulfasalazine in reducing inflammatory markers (TNFα, IL-6 and serum amyloid A), restoring concentrations of antioxidant agents (glutathione and cysteine) and attenuating colitis in murine models of both UC and CD [22].

A systematic review from Schneider et al. compared curcumin to a currently used biological anti-TNFalfa agent, Remicade, for the treatment of CD. Specifically, the authors highlighted the ability of different curcumin derivatives of suppressing the NF-kB signal pathway, and therefore inhibiting IL-6, IL-1 and TNF-a expression in IBD and in colorectal cancer. They went further, suggesting using curcumin as a complementary therapy to Remicade, particularly in patients with augmented IL-1 levels and, therefore, at high risk of loss of response to the biological agent [23]. In a subsequent randomized, double-blinded, multicentric study performed in Japan, a highly bioavailable curcumin derivative, Theracurmin^®^, was administered in a group of patients with mild-to-moderate CD (360 mg/day for 12 weeks) and compared to placebo. In the Theracurmin^®^ group, the authors observed a significant reduction in clinical disease activity and in endoscopic disease severity, as well as significant healing of anal lesions, along with a favorable safety profile [17]. 

Moreover, polyphenols are contained in a great number of other plants components, such as berries, barks, leaves and fruits. 

In a TNBS rat model of CD, a polyphenolic maqui berries (*Aristotelia chilensis*) extract was administered orally for 4 days before or after TNBS induction. The result was both a preventive and a curative effect of the polyphenolic extract with the inhibition of body weight loss and colon shortening, reduction in transmural inflammation and acceleration of mucosal healing [24]. Kolacek et al. treated 14 children suffering from CD with a 10-week course of a grapes and pine’s bark extract, called Pycnogenol, and they observed a reduction in the pro-inflammatory thromboxane B2 expression, which was augmented at baseline in CD patients compared to healthy controls [25]. An extract of olive leaves, containing oleuropeoside and other polyphenols, showed anti-inflammatory effect in intestinal mucosal samples from CD patients [26], whilst an apple polyphenol extract (APE) improved colon damage in a rat model of colitis by reducing COX-2, TNF-α, calpain and tissue transglutaminase mRNA expression [27]. 

*Boswellia serrata* and *Artemisia absinthium* extracts have been studied in CD in both preclinical and clinical trials. The first one is a gum resin obtained from the *B. Serrata* tree, which showed anti-inflammatory and antioxidant properties in rat models of ileitis; in one clinical trial on 102 patients with active CD, the resin resulted to be as effective as mesalamine in reducing the Chron’s Disease Activity Index (CDAI) score [18], whilst in a subsequent trial on 82 patients with quiescent CD, it was not effective in maintaining remission compared to placebo [19]. *Artemisia absinthium* extract, commonly known as wormwood, exerts its anti-inflammatory and antioxidative activity through two main derivatives, cardamonin and the flavonoid p7F. A randomized multicentric controlled trial conducted by Omer et al. on 40 CD patients during steroid tapering suggested that wormwood extract has a steroid-sparing effect with just 10% of patients in the wormwood group requiring the reintroduction of steroid after withdrawal, compared to 55% of the placebo group [20]. Later in 2010, they demonstrated that the same wormwood extract was able to reduce TNF-α levels in CD patients, which was a finding never obtained before for an herb in human models [21]. 

Finally, among phytochemicals, it is worth mentioning *Cannabis sativa* extracts, which are under the spotlight for their therapeutic potential in different chronic conditions. Specifically, cannabidiol, one of the main active phytocannabinoids contained in *Cannabis sativa*, is known to have antioxidant, anti-inflammatory and analgesic activities [28]. However, the other most represented cannabis’ constituent, Δ9-tetrahydrocannabinol, has psychedelic effects and the general concern about cannabis’ safety represents a main issue for its medical application on a large scale [29]. In CD, it seems that cannabis may have a potential therapeutic role, especially in symptom control, but research is still scarce with only a recent trial from Naftali et al. conducted on 56 CD patients by oral administration of either cannabis oil containing 160/40 mg/mL cannabidiol/tetrahydrocannabinol (CBD/THC) or placebo for eight weeks. Disease activity was evaluated before and after treatment with clinical (CDAI) and endoscopic scores (SES-CD). The results showed a significant improvement in CDAI scores in the cannabis group, while no significant changes in inflammatory parameters or endoscopic scores were registered [30]. Three other small clinical trials have been recently reviewed elsewhere [31].

## 3. Other Nutritional Approaches

### 3.1. Prebiotics and Probiotics

Dysbiosis has been proposed as an early pathogenetic drive in CD [32]. In a large pediatric CD population, Gevers et al. demonstrated how an abundance of bacteria, including Enterobacteriaceae, Pasteurellaceae, Veillonellaceae and Fusobacteriaceae, and a decreased number of Erysipelotrichales, Bacteroidales and Clostridiales strongly correlates with disease activity. 

Microbiome comparison between CD patients with and without antibiotic exposure indicated that antibiotic use contributes to the dysbiosis associated with CD [33]. According to the FAO/WHO definition, probiotics are ‘‘live microorganisms that confer a health benefit to the host when administered in adequate amounts’’ [34], while prebiotics are food components, more commonly oligosaccharides, which are not directly absorbed in the gut, but are converted by the intestinal microbiota into beneficial products. Natural food ingredients or dietary fibers found in some plants, such as fructo-oligosaccharides (FOS), galacto-oligosaccharides (GOS), lactulose and inulin, represent the most commonly used prebiotics [35]. 

Prebiotics are fibers, but the latter, to be defined as prebiotics, need scientific proof to attest this: they resist gastric pH, are fermented by the microbiota and are able to promote the selective growth of intestinal bacterial species associated with health benefits [36].

Regarding fiber, to date, the Food and Drug Administration (FDA) has approved two health claims: fiber from fruits, vegetables and whole grains may reduce the risk of certain types of cancer and their use is associated with reduced fat absorption (FDA, 21CFR101.76). The importance of prebiotics in CD pathophysiology is witnessed by the evidence that dietary fiber intake may alter the susceptibility to develop the disease. Indeed, Ananthakrishnan et al., in 2013, in a prospective study, analyzed data from 170,776 women, followed up over 26 years, who participated in the Nurses’ Health Study, evaluating associations between long-term intake of dietary fibers and the risk of incident CD or UC. Their data showed that a high-fibers diet, particularly from fruit, was associated with a 40% reduction in the development of CD. Nonetheless, few evidence support the role of prebiotics in controlling inflammation in IBD in general, and particularly in CD. A small open-labelled study involving 10 CD patients found that daily FOS supplementation (15 g) was able to significantly affect the composition of gut microbiota, by increasing fecal bifidobacteria, and could potentially modulate mucosal dendritic cell function. In this trial, they reported a significant reduction in disease activity (as measured by the Harvey Bradshaw index), but noted an increase in gastrointestinal symptoms, such as bloating and flatulence, following FOS supplementation [37]. Subsequently, a larger (*n* = 103) randomized placebo-controlled trial failed to demonstrate an improvement in CDAI scores in the FOS group. On the contrary, the authors recorded an increased severity of gastrointestinal symptoms and a higher withdrawal rate in the FOS arm as compared to placebo. This effect was likely to be secondary to the osmotic effect and bacterial fermentation secondary to FOS administration, adding to the concept that the worsening of functional IBS-like symptoms may overshadow the benefits of prebiotic supplementation in CD patients [38]. 

The role of probiotics in CD has been extensively reviewed elsewhere [39,40]. An overview of the mostly used probiotics in CD is presented in Table 2. 

While their beneficial role in UC has been well defined, their usefulness in CD therapy is still controversial. Indeed, a recent Cochrane metanalysis analyzing the efficacy of probiotics for the induction of remission in CD led the authors to conclude that “*the available evidence is very uncertain about the efficacy or safety of probiotics, when compared with placebo, for induction of remission in Crohn’s disease*”. However, the authors were able to include only two RCTs that met the eligibility criteria in the metanalysis (46 patients overall) given the lack of well-designed RCTs in this field of research. 

The most widely tested probiotic in CD is *Saccharomyces boulardii*, a non-pathogenic yeast, which seemed to have a promising role in CD. In a pioneering study by Plein and Hotz in 1993, Saccharomyces Boulardii showed to improve symptoms in patients with active CD (CDAI 193 ± 32), in combination with conventional therapy, and to further improve the frequency of bowel movements and the CDAI scores when administered, after the initial induction of remission phase, for an additional ten weeks, in patients with residual disease activity (CDAI 168 ± 59) [41]. These results were confirmed by another clinical trial conducted by Guslandi et al. in 2000: a total of 32 patients with CD in clinical remission (CDAI < 150) were randomly treated for six months with mesalamine plus a preparation of *Saccharomyces boulardii* (1 g daily) or with placebo (mesalamine alone). The combined preparation was able to reduce clinical relapses to 6.25% versus 37.5% in the control group [42]. Later in 2013, a bigger trial was conducted on 165 patients with steroid-induced remission who were randomly assigned to treatment with *Saccharomyces boulardii* (1 g/day) or placebo for 52 weeks. Unfortunately, no significant differences were found between the two groups, although a post hoc analysis indicated that *Saccharomyces boulardii* had a preventive role compared to placebo in non-smokers patients [43]. An increased number of adherent invasive *Escherichia coli* (AIEC) have been found in CD patients and AIEC is now believed to take part in CD’s pathogenesis. The probiotic *Escherichia coli Nissle 1917* (EcN) showed to inhibit AIEC invasion and to modulate cytokine secretion in an anti-inflammatory sense, when incubated with Caco-2 cells already infected with E. Coli LF82 [44]. *Bifidobacterium breve, Bifidobacterium longum,* and *Lactobacillus casei* are other probiotics studied in CD. In general, they showed to improve symptoms in patients with active CD, but none of them was able to achieve the suspension of corticosteroids in refractory patients or to obtain an improvement in inflammatory markers [45,48]. In 2000, Gupta et al. showed the efficacy of *Lactobacillus rhamnosus GG* in a small preliminary study on four children with mildly to moderately active CD. Patients who were given *Lactobacillus rhamnosus GG* in enteric-coated tablets twice a day for 6 months showed a significant improvement in the disease activity index and in the intestinal permeability, measured by a double sugar permeability test [46]. Regretfully, these results were not confirmed in a subsequent study on an adult population [47]. VSL#3 is a probiotic mixture of eight different bacterial strains (*Lactobacillus acidophilus*, *L. plantarum*, *L. casei*, *L. delbrueckii* subspecies *bulgaricus*, *Bifidobacterium breve, B. longum*, and *B. infantis* and *Streptococcus salivarius* subspecies *thermophilus*). Although it showed promising evidence in UC, VSL#3 was not effective in maintaining remission in patients with predominantly colonic CD and was even associated with a greater percentage of flares. Overall, according to present data, there are few possibilities that probiotics may have a role in inducing or maintaining remission in CD [49]. However, they seem to have a positive effect on symptom control, suggesting their possible application both in active disease for symptom relief and in quiescent disease for the control of post-inflammatory IBS-like symptoms. Nonetheless, an exciting chapter has recently opened up, potentially revolutionizing probiotic use in IBD care. Indeed, with the outstanding advancements in genetic engineering, we recently witnessed the development of bacterial/probiotic strains genetically engineered to either act as “intestinal biosensors” (detecting early inflammatory markers) or as “resident cell factories” of therapeutic molecules (improving drug delivery at the mucosal surface and avoiding systemic side effects). Different probiotic strains, indeed, have been engineered to deliver and produce in situ therapeutic substances: some express them constitutively, while others could react to externally administered substrates, increasing the overall efficiency of the system. Although most studies are still in the preclinical phase, few genetically engineered probiotics have been already tested in humans, in phase II clinical trials. Despite their great potential in preclinical models, however, this approach is still far from clinical transability due to safety issues regarding containment, specificity and toxicity. Furthermore, it may be challenging to deliver viable bacteria through probiotics due to poor survival and/or competition with the endogenous gut microbiome. This aspect would ideally depend on an individual IBD patient’s microbiome profile, theoretically leading to the personalization of therapy based on individual microbiome composition [50,51,52].

### 3.2. Micronutrients

A high proportion of patients with CD present signs of malnutrition due to macro- and micronutrient deficiency. While, for many years, the attention has been focused on macronutrient deficiency, nowadays, it is known that micronutrients also play a relevant role in CD pathophysiology. Furthermore, while macronutrient deficiency is generally found in advanced stages of CD, micronutrient deficiency can be insidiously present even in mild stages, however, with a considerable impact on disease course and complications. Nicholae-Catalin Mechie observed that about 85% of patients with IBD present with a deficit of micro- and macronutrients and show signs of malnutrition. 

Among micronutrients, vitamin D and zinc play a relevant role in the immune system functioning and their deficiency has been linked to infectious and autoimmune disease, including CD [3]. 

In CD disease, for years, vitamin D deficiency has been proposed as a consequence of pathology; however, in recent years, an increasing number of studies have observed how a different blood concentration of vitamin D can have a positive or negative impact on the disease [53]. The biological parameter used to define the vitamin D values is the serum concentration of 25OHD; it refers to a range below and above which the effects of a deficiency or an excess can occur. 

For the general population, the North American Institute of Medicine (IOM) considers sufficient a value of vitamin D 20 ng/mL (50 nmol/L), but it differs among different patients. In particular, with bone and kidney diseases, a value < 20 ng/ml (50 nmol/L) is defined as deficient, while a concentration from 20 to 30 ng/ml (50–75 nmol/L) corresponds to an insufficiency of vitamin D [54,55,56,57,58,59,60,61].

Finally, another study conducted on a multi-institutional cohort of 2809 patients with IBD concluded that vitamin D deficiency (<20 ng/mL or <50 nM) was associated with an increased risk of malignancy, and that each increment of 1 ng/mL in 25D was associated with an 8% reduction in the risk of developing colorectal cancer [53].

It is known that cells from the immune system are able to convert 25-hydroxyvitamin D to active 1,25-dihydroxyvitamin D and that they express the nuclear vitamin D receptors (VDR). As a consequence, their exposure to vitamin D promotes the maturation of antigen-presenting cells and the innate immune response to pathogens in general. From genome analysis has emerged that different VDR binding sites are significantly associated with loci linked to inflammatory immune-related disorders. The most noteworthy finding about the relationship between vitamin D and CD is that the gene encoding for NOD2 (nucleotide oligomerization domain protein 2), whose mutation is strongly associated with the development of CD, is a direct target of the VDR. Another VDR binding site was identified in the PTPN2 gene, a locus also associated with CD. These findings suggest that vitamin D deficiency is not only a consequence of CD, due to intestinal malabsorption of dietary vitamin D, but that it actively contributes to the pathogenesis of the disease; hence, vitamin D supplementation may suppress inflammation related to CD [62].

Vitamin D deficiency is very common in IBD patients in general and it has been suggested that serum levels of 25 (OH) D could represent an additional non-invasive marker for disease status’ characterization in IBD [3]. Caviezel et al., in 2018, compared IBD patients’ 25-OH-D3 levels to those of patients with IBS. They observed significantly decreased 25-OH-D3 levels in IBD and particularly in CD patients, where also a significant inverse association between C-reactive protein (CRP) and fecal calprotectin (FC) with 25-OH-D3 levels was observed [63].

In 2013, Yang et al. determined the dose of vitamin D needed to raise serum vitamin D levels above 40 ng/ml in patients with mild or moderate CD [64]. Eighteen patients were treated with Vitamin D3 oral supplementation at an initial dose of 1000 IU/day with an escalation every 2 weeks until the patients’ serum concentrations reached 40 ng/mL 25(OH)D3 or until they were taking the maximal dose of 5000 IU/day. Fourteen out of eighteen patients required the maximal dose of 5000 IU/d. After twenty-four weeks of supplementation, there was an effective increment in serum 25 (OH) D3 levels and both the CDAI and the quality-of-life scores, compared to the baseline, improved. Interestingly, plasma 25(OH)D < 20 ng/mL was associated with an increased risk of surgery and CD-related hospitalization compared with 25(OH)D levels ≥ 30 ng/mL. Furthermore, patients with CD who had initial 25(OH)D levels < 30 ng/mL, but then normalized their 25(OH)D, had a reduced likelihood of surgery compared with those who remained deficient [64]. This evidence suggests that the restoration of normal serum levels of vitamin D is useful in the management of patients with CD and thus should be recommended. A study published on Journal of Pediatric Gastroenterology and Nutrition concluded that a single high-dose vitamin D3 (300,000 IU) was as effective as weekly vitamin D3 supplementation for increasing vitamin D levels in pediatric IBD patients [65]. Since 60–70% of children with IBD have hypovitaminosis D, this is an additional risk to low bone mineral density when combined with corticosteroid therapy in patients with IBD. 

Zinc is an essential micronutrient absorbed in the small intestine that appears to have anti-inflammatory and antioxidative properties. In 2018, a meta-analysis of about 26,095 articles attested the involvement of zinc in autoimmune diseases; of these, about 60 studies observed a lower zinc concentration in subjects with autoimmune diseases than in healthy subjects [66]. 

Zinc deficiency has been shown to be more associated also with IBD hospitalizations. It has been observed that malnutrition is associated with ominous outcomes in IBD patients [67]. 

In rat models of colitis, zinc supplementation had a beneficial action on the intestinal barrier function and was associated with a reduced expression of pro-inflammatory cytokines. On the other hand, zinc deficiency exacerbated experimental colitis induced by DSS in rats. In two large prospective cohorts of women, the dietary intake of zinc was inversely associated with the risk of developing CD, but not UC [68].

In 2001, Sturniolo et al. evaluated the effect of zinc supplementation in 12 patients with quiescent CD and increased intestinal permeability, measured with the lactulose/mannitol ratio. They found that patients who were treated with zinc (110 mg three times a day) had an improved lactulose/mannitol ratio; their data suggested that zinc supplementation can resolve permeability alterations and may contribute to reduce the risk of relapses in patients with CD [69].

In 2017, Siva et al., in a study on 996 patients with IBD, including 773 patients with CD, observed that patients with reduced serum zinc levels were more likely to have adverse outcomes, such as increased incidence of hospitalizations, surgery and complications related to the disease; on the contrary, the normalization of zinc levels was associated with a better prognosis [70].

In summary, IBD is associated with a state of malnutrition of micro- and macronutrients. Among the micronutrients that seem to have a more important impact on the earliest stages of the disease are vitamin D and zinc. The deficiency of the latter seems to be associated with a worsening of the disease.

### 3.3. Polyunsaturated Fatty Acids

Polyunsaturated fatty acids (PUFAs) and particularly **ω**3-PUFA, abundantly present in fish oil and seed oil, have anti-inflammatory properties. Specifically, PUFAs inhibit the biosynthesis of important inflammation mediators, such as PGE2 and TNF-a, and, on the other hand, they act as substrates for the synthesis of anti-inflammatory molecules, known as resolvins and protectins. For this reason, PUFAs are seen as potential beneficial nutraceuticals in IBD.

While an increased consumption of **ω**6-PUFAs has been associated with an augmented risk of CD, a greater intake of **ω**3-PUFAs seem to have a protective role [71].

Dietary **ω**-3 PUFAs markedly attenuated colonic inflammation in a rat model of CD induced by 2,4,6-trinitrobenzene sulfonic acid (TNBS). Rats treated with intragastric **ω**3-PUFA (20 mg/kg/day) showed indeed a reduction in the disease activity index (DAI), colon macroscopic damage index (CMDI) and tissue damage index (TDI), along with a reduced expression of proinflammatory cytokines.

Recently, Klek et al. studied the effect of the addition of intravenous fish oil to parenteral nutrition in patients with UC, CD and chronic intestinal failure. In all these conditions, they observed a significant improvement in the inflammatory status during the treatment and, specifically in patients with CD, a reduction in CRP, erythrocytes sedimentation rate (ESR), procalcitonin, white blood cells (WBC), and IL-6 levels [72].

Despite these promising findings, clinical trials provided inconclusive or negative results, and a recent meta-analysis investigating the long-term effects of **ω**-3, **ω**-6 and total PUFA on IBD suggested that supplementation with PUFAs does not have a significant effect on the prevention or treatment of IBD.

This incongruence may be due to differences in study designs, the PUFA formulations used or patient compliance to the treatment and, therefore, more studies are needed to establish the actual role of PUFAs in CD.

### 3.4. Lactoferrin

Different properties, including antimicrobial and anticancer activity, have been related to lactoferrin (Lf) and, among the others, its immunomodulatory activity is of particular interest in CD. Lf is an iron-binding protein, belonging to the transferrin protein family, produced and released by mucosal epithelial cells and polymorphonucleated cells in different mammalian species, including humans, and widely present in the colostrum, breast milk and more other biological secretions. 

In the past years, Lf emerged as a key element in the mammalian immune system. Both bovine and human Lf are able to bind surface receptors on the T-cell line and the expression of Lf receptors has been reported in all T-cell subtypes. Lf is also involved in B/T-cells interaction and in the differentiation of immature B cells into efficient antigen presenting cells. Furthermore, Lf appears to possess both pro- and anti-inflammatory properties and to regulate cytokine levels. Additionally, fecal lactoferrin is a sensitive and specific biochemical marker of inflammation with higher levels registered in IBD patients compared with healthy controls and a positive correlation with disease severity [73].

In a rat model of DSS-induced colitis, the oral administration of Lf induced a significant increase in anti-inflammatory cytokines (IL-4 and IL-10) and a significant reduction in pro-inflammatory cytokines (TNF- α, IL-1β and IL-6), with a consistent improvement in disease severity [74]. Later, in 2014, Bertuccini et al. explored the ability of bovine Lf (b-Lf) to modulate the interactions between the intestinal epithelial cells and adherent-invasive *E. coli strain LF82* (AIEC), which is thought to be involved in CD pathogenesis. They found out that bacterial adhesion and invasion were normalized in AIEC infected cells when incubated with b-Lf. Moreover, they measured the mRNA expression of pro-inflammatory cytokines both in cultured cells and in biopsies taken from intestine of patients affected by CD and they found a significant reduction in TNF-α, IL-1β and IL-6 mRNA expression in the presence of b-Lf. Their data suggest that Lf exerts beneficial effects in CD both through antibacterial and anti-inflammatory mechanisms [75]. More recently, a recombinant human Lf, called VEN-120, was studied in two murine models of intestinal inflammation, the DSS-induced colitis model and the TNFΔARE/+ model of Crohn-like ileitis, by MacManus et al. They demonstrated that VEN-120 was able to reduce inflammation in both models of IBD, by increasing regulatory T cells in intestinal lamina propria and associated lymphoid tissue [76]. 

An interesting clinical experience was reported from Alexander et al., who described the case of a 22-year-old man with recent diagnosis of Crohn colitis. The patient was first treated with mesalazine, granulocyte apheresis and adalimumab, obtaining after 20 days of treatment an almost complete endoscopic remission with a marked improvement of his clinical status. After three months from the disease onset, he stopped the administration of adalimumab and started assuming 1 g of b-Lf per day without any other treatment. Subsequent examinations resulted in no sign of disease recurrence and, two years later, a colonoscopy showed almost complete mucosal healing. This case suggests the potential role of b-Lf in maintaining CD remission status, after an initial disease control is obtained by conventional therapy [77]. 

### 3.5. Palmitoylethanolamide

Palmitoylethanolamide (PEA) exerts both anti-inflammatory and antinociceptive effects, thus representing the prototype nutraceutical that can be used both in active and quiescent disease.

PEA is an endogenous N-acylethanolamine (NAE), isolated for the first time about 60 years ago and widely present in both animal and vegetal food (egg yolk, peanut oil and soy lecithin) and thought to have analgesic, anti-inflammatory and anti-angiogenic activities. It is biosynthesized endogenously as a result of the anti-inflammatory response, in conditions featured by neuronal damage and oxidative distress. PEA’s role has been more extensively studied in fibromyalgia syndrome and neurodegenerative disorders, while, more recently, its potential in counteracting intestinal inflammation has emerged.

One of PEA’s anti-inflammatory effects is the selective targeting of the S100B protein that is specifically expressed by enteric glial cells (EGCs). Beyond the well-known role of macrophages and neutrophils, EGCs also take part in the onset and progression of intestinal inflammation and an overexpression and secretion of the S100B protein has been reported in IBD patients. In mouse models of dextran sodium sulphate (DSS)-induced colitis, PEA as shown to significantly decrease ECGs activation and its effect appears to be mediated by the peroxisome proliferator-activated receptor-α (PPARα). Indeed, when PEA was co-administered with selective PPARα antagonists, its anti-inflammatory effects were almost completely abolished [78].

Moreover, PEA showed to inhibit colitis-associated angiogenesis, decreasing VEGF release and new vessels formation when administered in mouse models of DSS-induced colitis and in human derived colonic biopsies’ cultures [79]. These results, which are also elicited via a PPAR-α dependent mechanism, suggest that PEA may not only affect disease progression, but also its shift towards carcinogenesis, where neo-angiogenesis plays a key role [80].

In addition to its PPAR-α interactions, other plausible PEA targets are the cannabinoid receptors CB1/CB2 and the transient receptor potential vanilloid type-1 (TRPV1) ion channels. Specifically, Borrelli et al. demonstrated that the protective effect of PEA in a mouse model of colitis was associated with changes in TRPV1 channels, GPR55 and CB1 receptor mRNA expression. Interestingly, these results were obtained when PEA was administered both intraperitoneally and orally, leading the authors to consider its further use in humans [81]. In 2019, Couch et al. demonstrated that cannabidiol and PEA reduce colonic permeability both using in vitro and in vivo models. Given the association between IBD and increased gut permeability, these findings further support the use of PEA in IBD patients [82].

Regretfully, to date, no human trials have confirmed the results of these preclinical findings in CD. However, PEA has been tested in vivo in IBS patients. Specifically, in a double-blind, placebo-controlled and multicenter study on IBS patients, PEA resulted to be effective vs. placebo in reducing the severity of abdominal pain and discomfort [83]. These data suggest that PEA could be a promising approach for symptoms management also in IBD patients when an overlap IBS-like syndrome is present. 

## 4. Conclusions

Current knowledge indicates that several nutraceuticals show potential in countering inflammation in patients with Crohn’s disease and could represent an alternative treatment to conventional drugs in controlling residual functional symptoms in quiescent disease.

The use of nutraceutical supplements by IBD patients is steadily increasing and an estimated 50% of patients with IBD try complementary and alternative treatments, comprising nutraceuticals that are perceived as a safer and more natural approach in long-term IBD care [8,84]. 

However, current evidence of the efficacy of dietary supplements is mixed due to the suboptimal methodology of many human trials. Most nutraceutical use relies on preclinical studies showing a variable degree of efficacy in CD models, but further rigorous research is needed to translate the real benefits of these compounds in humans. Heterogeneity in terms of sample size, type of nutraceutical supplements, doses and duration of treatment and outcome measures give reason for the conflicting results produced for some nutraceuticals in terms of efficacy in IBD care. Overall, most studies are underpowered to detect a statistically significant effect. Cumulative results from systematic reviews and metanalyses could overcome this flaw; however, regrettably this is often not possible given to the high degree of heterogeneity in terms of study design and type of preparation (e.g., monotherapy vs. herbal mixture preparations, doses of purified supplements and duration of the intervention) [85]. The CDAI score is the most widely validated method to assess the efficacy of nutraceutical or any other therapeutic interventions in clinical trials. However, its use, while standard in some countries and health systems, is not always consistent throughout the world, further complicating the comparison of the results among interventional studies. Finally, particularly in quiescent CD, nutraceutical supplements could confer benefits to the host, not strictly related to the course of the underlying gastrointestinal condition, but rather to the health-related QoL, which are difficult to capture in short-term clinical trials. 

Phytochemicals, such as curcumin, Boswellia and Artemisia, have shown the most promising results in controlling gastrointestinal inflammation and maintaining clinical remission in RCTs. Additionally, zinc and vitamin D have been shown to be often deficient in patients with CD, often correlating with disease outcome; moreover, vitamin D deficiency represents an additional useful non-invasive marker for characterizing different disease activity states in patients with IBD. Lactoferrin through an antibacterial and/or anti-inflammatory mechanism exerts beneficial anti-inflammatory effects in Crohn’s disease. PEA has been shown to improve symptoms in IBS patients and in vitro and ex vivo evidence show a potential benefit in attenuating intestinal inflammation and increasing permeability. Finally, probiotic supplementation has not been shown to be effective in treating CD, but it could be beneficial in residual symptoms control (Table 3).

## 5. Discussion

As summarized in our manuscript, most nutraceuticals have been tested in animal models and more rigorous research is currently warranted in order to prove their real effectiveness in clinical settings. According to a survey involving IBD health-care professionals in Sweden, although most physician had a positive attitude toward alternative treatments, they felt that were ill-prepared to discuss complementary therapies that could aid in IBD patients care [86].

Hopefully, this review and other similar articles will encourage clinicians [87] to explore more accurately the use of nutraceuticals in this subset of patients and provide evidence-based studies to clarify the exact patients (quiescent vs. non-quiescent disease), doses and modality of administration that could actually benefit CD patients to properly counsel them and stimulate unbiased research with the same scientific rigor as any other area of pharmacology [88]. 

## Figures and Tables

**Table 1:** Phytochemicals tested in randomized controlled trails over placebo or standard. Treatment-observed outcomes and reported adverse events. AEs: Adverse events; HAMD score: Hamilton Depression Rating Scale; CDAI: Crohn’s Disease Activity Index.

Phytochemical (Dose and Duration)	Studied Population	Primary Endpoint	Outcomes	AEs	Authors (Year)
*Curcuma longa* derivative (Theracurmin^®^ 360 mg/day for 10 weeks)	30 patients with mild-to-moderate CD	Difference in CDAI improvement compared to placebo group	Lower CDAI score at week 12 compared to placebo (*p* < 0.005)	None	Sugimoto et al. (2020) [17]
*Boswellia serrata* extract, H15, 3.6 g/day for 8 weeks	102 patients with moderately active CD	Reduction in CDAI score	Non-inferiority compared to mesalamine 4.5 g/day orally	None	Gerhardt et al. (2001) [18]
*Boswellia serrata* (3 capsules of Boswelan twice a day for 52 weeks)	82 patients with quiescent CD	Proportion of patients who maintained remission throughout the 52 weeks	No significant difference compared to placebo (*p* = 0.85)	None	Holtmeier et al. (2011) [19]
*Artemisia absinthium* (3 capsules of Seda-Crohn^®^ twice a day for 10 weeks)	40 patients with mild-to-moderate active CD under corticosteroid treatment (starting tapering at week 2)	Decrease of 30% in CDAI score and decrease of >50% in total HAMD score from baseline	Significant reduction in CDAI score compared to placebo (*p* = 0.01) plus steroid-sparing effect	None	Omer et al. (2007) [20]
*Artemisia absinthium* (3 capsules of Seda-Crohn^®^ three times a day for 6 weeks)	20 patients with moderate active CD	70 point decrease in CDAI score or 50% decrease in HAMD score from baseline	Significant reduction in CDAI score and TNF- α levels compared to placebo (*p* = 0.05)	None	Krebs et al. (2010) [21]

**Table 2:** Overview of studies testing probiotics strains in CD. Note the high degree of heterogeneity in terms of probiotic formulations, duration of treatment, study design and target population (active vs. quiescent CD).

Probiotic Strain	Studied Population	Doses and Duration	Outcomes	Authors (Year)
*Saccharomyces boulardii*	CD patients (*n* = 20) suffering from diarrhea and augmented BEST index. After the first two weeks, patients randomly assigned to placebo or to S. Boulardii for additional 7 weeks, while the basic treatment was maintained.	▪ 250 mg t.i.d., initially for two weeks in addition to the basic treatment. ▪ 250 mg t.i.d. or placebo for additional 7 weeks, while the basic treatment was maintained.	Reduction in the frequency of bowel movements and in the BEST Index compared to baseline.	Plein and Hotz. (1993) [41]
*Saccharomyces boulardii*	CD patients (*n* = 32) in clinical remission (CDAI < 150) randomly treated with either mesalamine or mesalamine plus a preparation of Saccharomyces boulardii.	Six months with either mesalamine 1 g three times a day or mesalamine 1 g two times a day plus a preparation of Saccharomyces boulardii 1 g daily.	Clinical relapses as assessed by CDAI values were observed in 37.5% of patients receiving mesalamine alone and in 6.25% of patients in the group treated with mesalamine plus the probiotic agent.	Guslandi et al. (2000) [42]
*Saccharomyces boulardii*	CD patients (*n* = 165) in remission after treatment with steroids or salicylates, randomly assigned to groups given S. Boulardii or placebo.	S. Boulardii (1 g/day) or placebo for 52 weeks.	CD relapsed in 80 patients, 38 in the S boulardii group (47.5%) and 42 in the placebo group (53.2%): non-significant difference.	Bourreille et al. (2013) [43]
*Escherichia coli Nissle 1917*	Intestinal epithelial Caco-2 cell line infected with CD-Associated E. coli LF82.	Cells were co-infected with EcN (MOI of 10) after 3 h of monoinfection with strain LF82. After 6 h and 9 h of infection, the number of invasive bacteria was determined.	EcN showed an inhibitory effect on invasion by strain LF82.	Huebner at al. (2011) [44]
*Bifidobacterium breve, Bifidobacterium longum, Lactobacillus casei*	Active CD outpatients (*n* = 10), who failed to achieve remission with aminosalicylates and prednisolone, initiated on a symbiotic therapy, consisting of Bifidobacterium and Lactobacillus and Psyllium.	75 billion colony forming units [CFU] daily and psyllium 9.9 g daily.	▪ Improved clinical symptoms with both CDAI and IOIBD scores significantly reduced ▪ Not able to achieve suspension of corticosteroids or improvement in inflammatory markers.	Fujimori et al. (2007) [45]
*Lactobacillus rhamnosus GG*	Children with mildly to moderately active CD (*n* = 4) were given Lactobacillus GG.	10^10^ colony forming units (CFU) in enterocoated tablets twice a day for 6 months.	Significant improvement in clinical activity and intestinal permeability. Median pediatric CD activity index scores at 4 weeks 73% lower than baseline.	Gupta et al. (2000) [46]
*Lactobacillus rhamnosus GG*	Patients with moderate-to-active CD (*n* = 11) randomly assigned to receive either Lactobacillus GG or placebo.	10^9^ CFU twice daily or placebo for six months.	No significant difference in frequency of relapses between the two groups.	Schultz et al. (2004) [47]

**Table 3:** Other nutraceuticals tested in CD, their proposed mechanisms of action and preclinical evidences supporting their efficacy in populations of CD patients. ECG (Enteric Glial Cells); S100B (S100 Calcium—binding protein B); TRPV1 (Transient Receptor Potential Vanilloid 1); PPARα (Peroxisome Proliferator—Activated Receptor Alpha); VEGF (Vascular Endothelial Growth Factor); DSS (Dextran Sodium Sulphate); UC (Ulcerative Colitis); DNBS (Dinitrobenzenesulfonic acid); RCT (Randomized Controlled Trial); PGE2 (Prostaglandin E2); VDR (Vitamin D Receptor). Note: phytochemicals are not included because they are summarized in Table 1.

Nutraceutical	Target Population	Mechanism(s) of Action	Studied Models	Ref
Palmitoyl- ethanolamide (PEA)	Active CD (anti-inflammatory and antiangiogenic effects)	Anti-inflammatory effects: ▪ PPARα-mediated downregulation of proinflammatory cytokines, EGCs and mast cells ▪ Decreased colonic permeability ▪ Inhibition of colitis-associated angiogenesis	Murine models of TBNS and DSS-induced colitis, colonic biopsies deriving from UC patients and primary cultures of mouse and human EGCs	Esposito et al., 2014 [78] Sarnelli et al., 2016 [80] Borrelli et al., 2015 [81]
	Clinically quiescent CD (analgesic properties)	Analgesic effects: Downregulation of TRPV1 channels “Entourage” effects on endocannabinoid system	Analgesic effects in IBS (Double-Blind, Placebo controlled RCT)	Cremon et al., 2017 [83]
Lactoferrin (Lf)	Active CD	Anti-inflammatory effects: ▪ Increase in anti-inflammatory cytokines (IL-4 and IL-10) and regulatory T cells and decreased pro-inflammatory cytokines (TNF- α, IL-1β and IL-6)	Murine models (DSS-induced colitis and TNFΔARE/+ model of Crohn-like ileitis), in vitro and ex vivo biopsies deriving from CD patients	Togawa et al., 2002 [74] MacManus et al., 2017 [76]
	Maintenance of remission (?)	Antibacterial effect: Inhibition of bacterial invasion	In vitro models	
Vitamin D3	Active CD	Effect on genome: ▪ VDR binding sites on genetic loci linked to CD (NOD2 and PTPN2)	Interleukin-10 knockout mice, CD risk of surgery (prospective cohort study on effect of vitamin D normalization)	White, 2018 [62]
	Mild-to -moderate CD	Immunomodulatory effect: Promotes the maturation of antigen-presenting cells and innate immune response to pathogens through VDR	Therapeutic effect of vitamin D supplementation in CD (Open-label prospective clinical trial)	Caviezel et al., 2018 [63]
Zinc	Clinically quiescent CD (Prevention of relapses and complications)	Anti-inflammatory effect: ▪ Reduced expression of pro-inflammatory cytokines ▪ Improvement of intestinal barrier function	Murine models of DSS-induced colitis Zinc intake and risk of CD (prospective cohort study)	Ananthakrishnan et al., 2015 [68] Sturniolo et al., 2001 [69]
Poly- unsaturated fatty acids (PUFAs)	Mild-to-moderate CD (Chronic intestinal failure associated with CD)	Anti-inflammatory effect: ▪ Inhibition of pro-inflammatory mediators as PGE2 and TNF-alfa ▪ Substrates for the synthesis of anti-inflammatory molecules, resolvins and protectins	TNBS-induced colitis in mice Effect of intravenous fish oil addition on parental nutrition in CD patients (retrospective multicentric analysis)	Siva et al., 2017 [70]; Scoville et al., 2019 [71]
